# High-order brain functional network for electroencephalography-based diagnosis of major depressive disorder

**DOI:** 10.3389/fnins.2022.976229

**Published:** 2022-08-09

**Authors:** Feng Zhao, Hongxin Pan, Na Li, Xiaobo Chen, Haicheng Zhang, Ning Mao, Yande Ren

**Affiliations:** ^1^School of Computer Science and Technology, Shandong Technology and Business University, Yantai, China; ^2^Department of Radiology, Yantai Yuhuangding Hospital, Yantai, China; ^3^Department of Radiology, The Affiliated Hospital of Qingdao University, Qingdao, China

**Keywords:** electroencephalography, brain functional networks, major depressive disorder, high-order brain functional network, disease classification

## Abstract

Brain functional network (BFN) based on electroencephalography (EEG) has been widely used to diagnose brain diseases, such as major depressive disorder (MDD). However, most existing BFNs only consider the correlation between two channels, ignoring the high-level interaction among multiple channels that contain more rich information for diagnosing brain diseases. In such a sense, the BFN is called low-order BFN (LO-BFN). In order to fully explore the high-level interactive information among multiple channels of the EEG signals, a scheme for constructing a high-order BFN (HO-BFN) based on the “correlation’s correlation” strategy is proposed in this paper. Specifically, the entire EEG time series is firstly divided into multiple epochs by sliding window. For each epoch, the short-term correlation between channels is calculated to construct a LO-BFN. The correlation time series of all channel pairs are formulated by these LO-BFNs obtained from all epochs to describe the dynamic change of short-term correlation along the time. To construct HO-BFN, we cluster all correlation time series to avoid the problems caused by high dimensionality, and the correlation of the average correlation time series from different clusters is calculated to reflect the high-order correlation among multiple channels. Experimental results demonstrate the efficiency of the proposed HO-BFN in MDD identification, and its integration with the LO-BFN can further improve the recognition rate.

## Introduction

Major depressive disorder (MDD) is a kind of common brain disease that is characterized by persistent and significant low mood, slow thinking, and cognitive function impairment (Holma et al., 2014; LeMoult and Gotlib, 2019; Liu et al., 2020). In the statistics of the World Health Organization (WHO), MDD has become the second largest serious disease in the world (Melchior et al., 2013) and has brought a heavy burden on patients and their families (Zhang et al., 2019). According to medical researches, the accurate early identification of MDD is important, and it can not only effectively relieve the pain of the patients, but also directly reduce the tragedy of suicide (Cipriani et al., 2018; Grunebaum et al., 2018). However, early neuroimaging-based MDD diagnosis is very challenging, because the changes of brain functional connectivity (FC) are considerably complicated. Electroencephalography (EEG) of high temporal resolution (Babajani and Soltanian-Zadeh, 2006) can well describe the temporal evolution of complex FC during brain activity and thereby becomes the best choice for MDD research.

Brain functional network (BFN) constructed based on EEG has been widely used in the diagnosis of brain diseases (Wang et al., 2015; Li et al., 2020). Since brain activity is dynamic in nature, some studies have shown that the dynamic change of FC over the whole scanning time may be the intrinsic feature of brain function (Damaraju et al., 2014; Cohen and D’Esposito, 2016; Kudela et al., 2017). Many studies try to describe the dynamic changes of FC between channels by using sliding windows to construct BFN and the relationship between these dynamic changes and brain diseases (Wee et al., 2016; Guo et al., 2017; Sun et al., 2019; Zhang et al., 2021). Sun et al. (2019) constructed BFN based on EEG signals by sliding windows, confirming that MDD had abnormal cognitive processing. Zhang et al. (2021) used sliding windows to construct BFN based on EEG signals, and the results showed that the brain regions of MDD patients were significantly altered.

**FIGURE 1:**
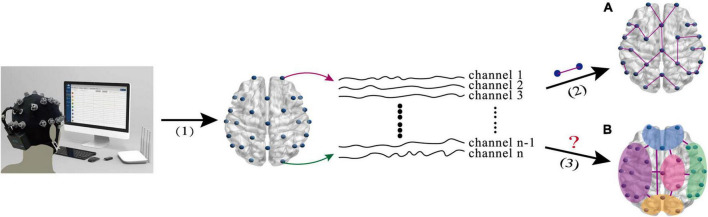
The intuitive explanation of brain network research based on EEG signals. **(A)** The low-order FC (LO-FC) between two channels. **(B)** High-order FC (HO-FC) among channels. (1) EEG signal acquisition(1) EEG signal acquisition, (2) calculation of correlation between two channels, and (3) calculation of correlation among multiple channels.

Although the aforementioned EEG-based BFN helps us to understand the brain activities of the MDD patients, most of them only reflect the low-order FC (LO-FC) between two channels (as shown in Figure 1A), ignoring the fact that high-order FC (HO-FC) among channels could also be changed for MDD patients (as shown in Figure 1B). For the ease of description, we call BFN based on conventional LO-FC as low-order BFN (LO-BFN). In essence, brain activity is complex, and the HO-FC usually contains more abstract information than the LO-FC, and it helps to reveal high-level and more complex interaction information (Chen et al., 2016). Therefore, it is of clinical significance to investigate effective methods of constructing a high-order BFN (HO-BFN) that better reflects the complex interaction among multiple channels and simulates the mechanisms of the deep brain, providing rich discriminative information for the diagnosis of mental disorders (Plis et al., 2014; Chen et al., 2016; Zhang et al., 2016).

In this work, we propose a novel method to construct HO-BFN for MDD classification. Specifically, the entire EEG signals of a subject are divided into multiple overlapping time series by sliding windows, and the correlation between two channels within one window is computed as the LO-FC. The network constructed by the LO-FC is called the LO-BFN, reflecting the dynamic change of FC throughout the whole scanning time. Significantly different from the LO-BFN, each vertex of the HO-BFN represents one pair of channels, and each edge represents the correlation between the channel pairs. In this way, the HO-BFN involves more channels and can reveal high-level and more interaction among brain regions.

It is noted that the scale of the HO-BFN is very huge, and it may suffer from the dilemmas of high dimensionality and small size samples. The main reason lies in the fact that the scale of HO-BFN will increase exponentially when the number of EEG channels increases. To address the issue, we introduce hierarchical clustering (Yu et al., 2015) to construct HO-BFN. In other words, similar LO-FC time series are clustered into one group, and the average LO-FC time series are computed for each group. After that, the HO-BFN is constructed based on the correlation between the two groups. As a result, the HO-BFN constructed by hierarchical clustering can not only reduce computing time and memory requirements, but also reflect the HO-FC among multiple channel pairs (more than four channels) and capture more useful and complex information.

In summary, the main contributions of the paper line are twofold: (1) A HO-FC representation strategy is proposed to capture high-order interactions among multiple channels of EEG signals. In fact, the HO-BFN is used to characterize the complex interactions among brain regions, and it has been applied in fMRI and achieved good results (Zhao et al., 2018). However, to the best of our knowledge, few studies have used EEG-based HO-BFN to reveal the complex interactions among EEG channels. (2) The HO-BFN is constructed in both time and frequency domains based on the “correlation’s correlation” strategy. Specifically, we first compute the correlation between two channels to obtain the LO-BFN, and then the HO-BFN is subsequently derived by computing the correlation between each pair of channels from the LO-BFN. Then, we further apply HO-BFN to computer-aided diagnosis for MDD. The experimental results show that HO-BFN provides complementary identification information to the LO-BFN and that combining HO-BFN and LO-BFN can further improve the accuracy of MDD diagnosis.

## Materials and data preprocessing

The EEG data used in this study came from the publicly available Multi-modal Open Dataset for Mental-disorder Analysis (MODMA) dataset (Cai et al., 2020). It included 24 patients with MDD (12 male and 12 female) and 29 normal controls (NC) (20 male and 9 female). The MDD group was 16–52 years old, and the NC group was 19–51 years old; all the subjects were right-handed, and their education level was primary school or above. In addition, MDD patients had a health Questionnaire 9-item (PHQ-9) (Spitzer et al., 1999) score greater than or equal to 5 and had not received psychotropic medication for 2 weeks. Table 1 shows the statistics of the subjects.

**TABLE 1:** Demographic information of the subjects.

	MDD	NC	*p*-value
Gender (M/F)	12/12	20/9	0.1600*^a^*
Age (mean ± SD)	30.9 ± 21.1	30.9 ± 20.1	0.9880*^b^*
PHQ-9 (mean ± SD)	18.3 ± 7.3	2.6 ± 2.6	0.0000*^b^*
GAD-7 (mean ± SD)	13.4 ± 11.4	2.1 ± 4.9	0.0000*^b^*

MDD, major depression disorder; NC, normal control; M, male; F, female; PHQ-9, Patient Health Questionnaire-9item; GAD-7, generalized anxiety disorder-7. p^*a*^: Statistical significance level was calculated by χ^2^-test; p^*b*^: Statistical significance level was obtained by two-sample, two-tailed t-test.

In data acquisition, 128-channel HydroCel Geodesic Sensor Net and Net Station acquisition software were used to record EEG signals for five minutes. Taking Cz as the reference, the sampling rate was 250 Hz. In order to reduce the interference of EEG data, the subjects were required to close their eyes and keep awake to avoid any unnecessary eye movement, saccade, and blink. The collected EEG data were filtered by 0.1–40 Hz and inhibited by 48–52 Hz to eliminate the data interference caused by baseline drift and electrical interference. The processed data was then re-referenced against REST (Yao, 2001). Finally, after the above steps, some high-power content was contained in the remaining data points and some EEG epochs were removed by the Artifact Subspace Reconstruction (ASR) plugin (Chang et al., 2018; Pion-Tonachini et al., 2018). In this study, theta (4–8 Hz), alpha (8–13 Hz), and beta (13–40 Hz) bands calculated by fast Fourier transform were selected in the frequency domain, which had been proved to be much distinct in the identification of depression (Nyström et al., 1986; Knott et al., 2001; Jaworska et al., 2012).

## Methods

**FIGURE 2:**
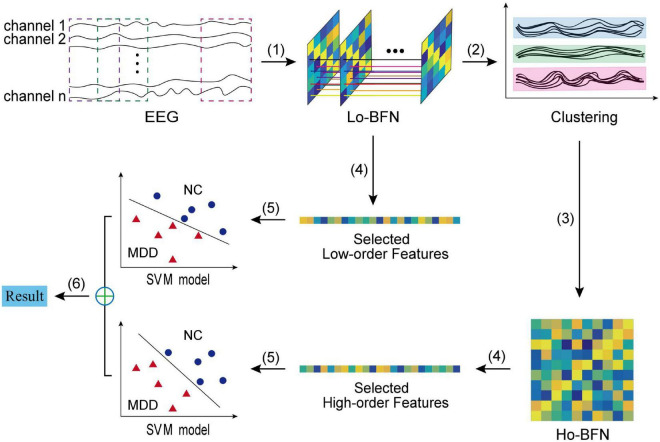
The flowchart of the proposed BFN classification framework, including six main steps: (1) constructing LO-BFN; (2) clustering the time series; (3) constructing HO-BFN; (4) feature selection; (5) constructing SVM model; and (6) classification fusion.

Figure 2 shows the overall pipeline of our method, which includes six steps: (1) constructing LO-BFN by sliding window; (2) clustering all low-order correlation time series; (3) constructing HO-BFN by calculating the correlation between clusters; (4) selecting and extracting features from each constructed BFN; (5) constructing support vector machines (SVMs) based on the selected features in both LO-BFN and HO-BFN; and (6) fusing the decision scores of multiple SVMs to predict whether each subject is MDD or NC.

### Construction of low-order BFN

In order to construct LO-BFN, we first use the sliding window to divide the entire EEG signals into *H* = [(*M*−*W*)/*s*] + 1 overlapping windows, where *M* is the image volume during the entire scan period, and *W* and *s* are the window width and step size of the sliding window, respectively. Then, we calculate the correlation between $x_{i}^{l}\left(h\right)$ and $x_{j}^{l}\left(h\right)$, where $x_{i}^{l}\left(h\right)$ and $x_{j}^{l}\left(h\right)$ denote the *i*-th and *j*-th channels under the *h*-th window of the *l*-th subject, respectively.

In the frequency domain, we use the phase lag index (PLI) (Stam et al., 2007; Peraza et al., 2012) to calculate the channel correlation, which is robust to volume conduction artifacts. The PLI is denoted as:


(1) $$C_{ij}^{l}=\left|\frac{1}{N}\sum_{n=1}^{N}sign\left(\varphi_{i}^{l}\left(t_{n}\right)-\varphi_{j}^{l}\left(t_{n}\right)\right)\right|$$

where *N* is the sample number, *sign* is the sign function, and $\varphi_{i}^{l}\left(t_{n}\right)-\varphi_{j}^{l}\left(t_{n}\right)$ is the phase synchronization of channels $x_{i}^{l}$ and $x_{j}^{l}$ at time *t*_*n*_. Among them, φ*^l^*(*t*_*n*_) can be obtained by analyzing the signal based on Hilbert transform (Bruns, 2004).

On the other hand, in the time domain, we calculate the channel correlation by using the Pearson correlation coefficient (PCC) (Eslami and Saeed, 2018) as follows:


(2) $$C_{ij}^{l}=corr\left(x_{i}^{l},x_{j}^{l}\right)$$

**FIGURE 3:**
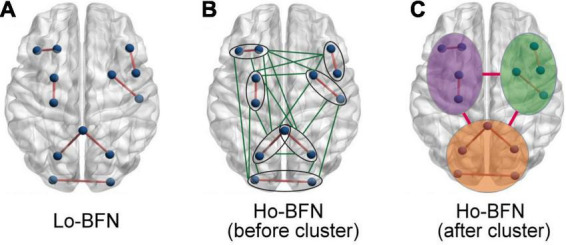
Schematic diagram of different BFNs. **(A)** Schematic diagram of the brain region of LO-BFN. **(B)** Schematic diagram of brain regions of HO-BFN before clustering. **(C)** Schematic diagram of brain regions of HO-BFN after clustering.

Therefore, for the *l*-th subject, the *h*-th subnetwork of LO-BFN is constructed as $G_{L}^{l}\left(h\right)=\left(\left\{x_{i}^{l}\left(h\right)\right\},\left\{C_{ij}^{l}\left(h\right)\right\}\right)$ (as shown in Figure 3A), where $\left\{x_{i}^{l}\left(h\right)\right\}$ is vertices and $\left\{C_{ij}^{l}\left(h\right)\right\}$ is the weights of the edges connecting the *i*-th and *j*-th nodes. Then, we construct *H* subnetworks to form LO-BFN $G_{L}^{l}=\left[G_{L}^{l}\left(1\right),G_{L}^{l}\left(2\right),\ldots ,G_{L}^{l}\left(H\right)\right]$ for each subject, which describes the change in FC strength of all channel pairs over time.

### Construction of high-order BFN

In order to capture high-level FCs, we adopt the strategy of “correlation’s correlation”. That is, based on the LO-BFN, the PCC is used to calculate the correlation between the LO-FC time series of the *l*-th subject, which is called HO-FC, denoted as:


(3) $$H_{ij,pq}^{l}=corr\left(C_{ij}^{l},C_{pq}^{l}\right)$$

where $C_{ij}^{l}$ is the LO-FC time series between the *i*-th and the *j*-th channels of the *l*-th subject and $C_{pq}^{l}$ is the LO-FC time series between the *p*-th and the *q*-th channels of the *l*-th subject. Therefore, $H_{ij,pq}^{l}$ can represent the HO-FC among the four channels of the *l*-th subject at most, that is, the correlation between the FC between the *i*-th and *j*-th channels and the FC between the *p*-th and *q*-th channels. Physiologically, difference in FC among different channels in MDD patients and healthy individual subjects can be used to identify MDD.

If we have 128 channels in our study, the dimensionality of the constructed LO-BFN is 128 × 128. Thus, a large-scale HO-BFN will be constructed (as shown in Figure 3B) in Eq. 3; that is, the dimensionality is (128 × 128)^2^, and the constructed network contains at least thousands of nodes and millions of edges. It is a critical problem that the dimensionality is too large, and it will introduce high computational complexity for the subsequent feature extraction and selection procedures. Besides, the generalization performance of the HO-BFN learning system may also degrade.

To this end, we will reduce the network dimensionality by clustering the LO-FC time series. Specifically, the LO-FC time series of the subjects are clustered into different clusters to find the potential interaction patterns. Then, the HO-FC between the respective average LO-FC time series in clusters is calculated. Compared with the previous large-scale HO-BFN, the dimensionality of the HO-BFN constructed in this way is greatly reduced (as shown in Figure 3C). This method not only preserves important interactive information, but also avoids the problems of high computational complexity and low generalization performance.

In order to ensure that the clustering results in different subjects are consistent, the FC matrices of all subjects are first accumulated together, so that to connect the time series of LO-FC in the same channel pairs in all subjects into a long vector. That is, the long vector connected by the time series ${\left\{C_{ij}^{l}\right\}}_{1\leq l\leq R}$ of the LO-FC between the *i*-th and *j*-th channels of all subjects is $C_{ij}=\left[C_{ij}^{1},C_{ij}^{2},\cdots ,C_{ij}^{R}\right]$, where *R* is the number of subjects.

After the *C*_*ij*_ is obtained, we divide it into *k* clusters by hierarchical clustering. The cluster centers are calculated by averaging all the FC long vectors in the *k*-th cluster of the *l*-th subject as the following Eq. 4:


(4) $${\tilde{C}}_{k}^{l}=\frac{\sum_{C_{ij}\in \omega_{k}}C_{ij}^{l}}{\left|\omega_{k}\right|}$$

where |ω_*k*_| denotes the total number of FC long vectors in cluster *k*. Finally, the PCC between the two clusters ${\tilde{C}}_{k1}^{l}$ and ${\tilde{C}}_{k2}^{l}$ of the *l*-th subject is calculated as follows:


(5) $${\tilde{H}}_{k1,k2}^{l}=corr\left({\tilde{C}}_{k1}^{l},{\tilde{C}}_{k2}^{l}\right)$$

Finally, we obtain a small-scale HO-BFN $G_{\tilde{H}}^{l}=\left(\left\{{\tilde{C}}_{k1}^{l}\right\},\left\{{\tilde{H}}_{k1,k2}^{l}\right\}\right)$, taking $\left\{{\tilde{C}}_{k1}^{l}\right\}$ as vertices and $\left\{{\tilde{H}}_{k1,k2}^{l}\right\}$ as the weights of edges.

### Feature extraction, selection, classification, and fusion

Both LO-BFN and HO-BFN of the *l*-th subject, i.e., $G_{L}^{l}$ and $G_{\tilde{H}}^{l}$, are used for the subsequent classification. Due to the possible phase mismatch of all FC matrices of LO-BFN in each subject, the dynamic characteristics of different subjects do not completely correspond. Therefore, to avoid this situation, we calculate the average FC matrix of each subject’s LO-BFN as the low-order feature. For the ease of computation, we vectorize the averaged LO-FC matrix of the *l*-th subject into $f_{L}^{l}$, which is called the low-order feature vector. Similarly, we vectorize the FC matrix ${\tilde{H}}^{l}$ of HO-BFN of the *l*-th subject to $f_{\tilde{H}}^{l}$ as the high-order feature vector.

Both low-order feature vectors and high-order feature vectors have a large number of features, introducing irrelevant or redundant information for subsequent MDD classification. Therefore, we use *t*-test and Least Absolute Shrinkage and Selection Operator (LASSO) (Tibshirani, 2011) methods to select features for high-order and low-order feature vectors, which can effectively remove redundant features. Specifically, we first perform *t*-test on both the low-order feature vector *f*_*L*_ and the high-order feature vector $f_{\tilde{H}}$ on the training set and select the features that are significantly different from *f*_*L*_ and $f_{\tilde{H}}$ as preliminary features, denoted ${\bar{f}}_{L}$ and ${\bar{f}}_{\tilde{H}}$, respectively.

Then, we use LASSO to further remove redundant features and select the features most related to MDD. Let *I^l^* be the labels for the *l*-th subject. Specifically, if the *l*-th subject is MDD, *I^l^* = −1; if the *l*-th subject is NC, *I^l^* = + 1; and α is set to be the weight vector for feature selection. The objective of LASSO is defined as:


(6) $$\min_{\alpha }\frac{1}{2}\sum_{l=1}^{N}{||I^{l}\left\langle -{\bar{f}}^{l},\alpha \right\rangle ||}_{2}^{2}+\lambda {||\alpha ||}_{1}$$

where ⟨⋅,⋅⟩ is the inner product operator and λ is the regularization parameter. For simplicity, let ${\bar{\bar{f}}}_{L}$ and ${\bar{\bar{f}}}_{\tilde{H}}$ denote selected the final feature sets from the feature vectors ${\bar{f}}_{L}$ and ${\bar{f}}_{\tilde{H}}$, respectively.

Finally, the MDD is classified by using SVM with a linear kernel in this paper. Herein, we train two linear SVM classifiers by using both ${\bar{\bar{f}}}_{L}$ and ${\bar{\bar{f}}}_{\tilde{H}}$ features, respectively. The final results are obtained by fusing the decision scores of two SVM classifiers by linear combination. Among them, we set the weight β ∈ [0.1,0.2,…,0.9] for each SVM. The weights of the *F* classifiers to be fused are set to be β_1_,β_2_,…,β_*F*_ and satisfy β1+β2+…+β=F1.

## Experiments results

To evaluate the effectiveness of our proposed method, we analyze the impact of clustering parameters on HO-BFN. The classification ability of LO-BFN, HO-BFN, and their fusion for MDD is evaluated by six different indicators, i.e., accuracy (ACC), sensitivity or true positive rate (TPR), specificity or true negative rate (TNR), precision or positive predictive value (PPV), negative predictive value (NPV), and F1 score. Finally, we conduct feature analysis to understand the role of each channel in MDD diagnosis.

Furthermore, we adopt a nested ten-fold cross-validation (CV) strategy consisting of two nested loops to evaluate the effectiveness of our proposed method. In the outer loop, all data are randomly divided into ten subsets of roughly the same size, where one subset is selected as the testing set, while the other nine subsets are selected as the training set. In the inner loop, the data of the training set are merged and redivided into ten subsets of similar size, nine of which are used to adjust the hyper-parameters and one for model evaluation. We report the average accuracy of classification results across the ten-fold CV. Then, in order to avoid any possible bias in fold selection, this procedure is repeated 10 times, with a different random partitioning of samples each time. Finally, the average accuracy of 10 repetitions is reported. Since the performance of our method also depends on hyper-parameters, such as *W* and *s* in sliding window, *k* in clustering, *p* in *t*-test, λ in LASSO, and *c* in SVM model. The optimal hyper-parameters can be determined when the average classification accuracy reaches its optimum. In our experiment, we fix the size of the sliding window, i.e., *W* = 10000, *s* = 1000, and determine the optimal values of other parameters within the following range: *k* ∈ [100, 200, …, 800], *p* ∈ [0.01, 0.02, …, 0.05], λ ∈ [0.1, 0.2, …, 0.9], and *c* ∈ [2^−4^, 2^−3^, …,2^4^].

### The clustering effectiveness on high-order BFN

**FIGURE 4:**
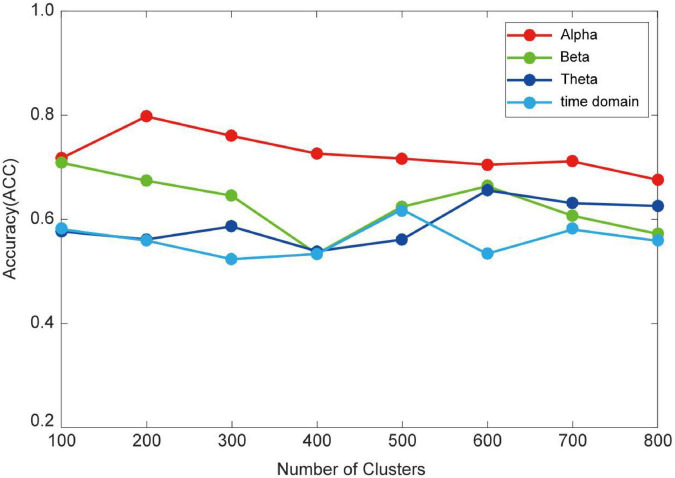
Recognition accuracy of HO-BFN with different number of clusters.

To reduce the computational complexity of the HO-BFN, the hierarchical clustering is employed to construct HO-BFNs. The hyper-parameter *k* in hierarchical clustering indicates the cluster number, and it has a crucial influence on the constructed HO-BFN, and further affects the final classification results. In the experiment, we optimize HO-BFN by adjusting the clustering number *k*. Figure 4 shows the classification results when *k* takes different numbers.

As can be seen from Figure 4 that for the clustering parameter *k*, when the time domain takes the value of 500, alpha takes the value of 200, beta takes the value of 100, and theta takes the value of 600, the HO-BFN generates a relatively satisfactory classification result. The ACC is greatly affected by *k*; i.e., classification performance is very sensitive to clustering parameters. Different HO-BFNs have different performances, indicating that different HO-BFNs contain different levels of MDD diagnosis information. Therefore, we can conclude that it is necessary to select *k* carefully toward a better understanding of dynamics in brains.

### Comparison of major depressive disorder diagnosis using different brain functional networks

In this subsection, we further study how HO-BFN contributes to MDD diagnosis. We construct and analyze HO-BFN from the time domain and alpha, beta, and theta bands in the frequency domain, respectively. In the experiment, we train and test the classifiers of LO-BFN and HO-BFN, respectively, and determine the parameter combination that can produce the best ACC.

Due to the complex connections in the brain, it is difficult to fully capture the relationship between different brain regions through a single type of BFN. In order to further improve the classification performance, we adopt the linear fusion of the SVM integrated decision score to combine LO-BFN and HO-BFN (Zhao et al., 2020) and analyze their fusion performance. In addition, we also believe that different BFNs constructed by the three bands in the frequency domain can reflect FC between channels from different views, which are complementary. Therefore, we further linearly fuse the decision scores of BFNs in three bands. Table 2 shows the classification performance of different BFNs, and the best classification results are highlighted in bold.

**TABLE 2:** MDD classification using different BFNs.

Network	ACC (%)	TPR (%)	TNR (%)	PPV	NPV	F1 (%)
Alpha-LO	60.67	59.17	60.83	64.81	54.86	61.78
Alpha-HO	79.78	80.83	78.33	82.25	77.08	81.48
Alpha-Fu	83.98	84.34	82.67	85.80	81.39	85.01
Beta-LO	64.52	66.17	62.17	68.17	59.96	67.07
Beta-HO	70.90	71.50	69.83	74.39	66.68	72.71
Beta-Fu	76.63	77.83	74.00	78.82	73.24	78.25
Theta-LO	69.63	71.50	68.50	73.43	66.29	72.23
Theta-HO	65.58	64.50	67.17	70.46	60.68	67.13
Theta-Fu	77.65	80.83	74.00	79.44	76.04	80.08
Frequency domain-Fu	**86.62**	**89.17**	**82.67**	**86.48**	**86.19**	**87.77**
Time domain-LO	59.16	60.50	58.17	63.75	54.56	61.94
Time domain-HO	61.23	62.67	60.33	65.85	56.85	64.11
Time domain-Fu	65.79	69.67	60.33	68.17	61.92	68.78

LO = LO-BFN; HO = HO-BFN; Fu, the fusion of LO-BFN and HO-BFN. For example, alpha-LO means the LO-BFN in the alpha band, and note that frequency domain-Fu means the fusion of all BFNs of three bands in the frequency domain. Values highlighted in bold indicate the best results.

From Table 2, we can draw the following conclusions: (1) BFNs constructed in different ways have different performance, implying that each BFN provides meaningful and various information for MDD identification; (2) fusing LO-BFN and HO-BFN is better than that of a single network, and the ACC is relatively increased in 4%, indicating that LO-BFN and HO-BFN are complementary to each other in classifying MDD; (3) the performance of each brain network in the frequency domain is obviously better than that of the BFNs in the time domain, indicating that it is more effective to extract features in the frequency domain.

### The most discriminative features for major depressive disorder diagnosis

To identify the most discriminative features in MDD diagnosis, we select ten frequently selected LO-FC features and two frequently selected HO-FC features based on the *t*-test and LASSO regression ten-fold cross-validations of ten times. The higher selection frequency of FC indicates stronger reliability and discriminative ability.

Similar to previous studies (Bian et al., 2014), we divide the brain into five regions, i.e., frontal (F), left temporal (LT), central (C), right temporal (RT), and posterior (P). The frontal region is further divided into left frontal (LF) and right frontal (RF), the central region is further divided into left central (LC) and right central (RC), and the posterior is further divided into left posterior (LP) and right posterior (RP). Table 3 shows the details of these brain regions.

**TABLE 3:** Brain regions corresponding to channels of interest.

Brain area	Channels
Frontal (F)	E2, E3, E4, E5, E9, E10, E11, E12, E15, E16, E18, E19, E22, E23, E24, E26, E27, E123, E124
Left temporal (LT)	E28, E33, E34, E35, E39, E40, E41, E45, E46, E47, E50, E51, E52, E58
Central (C)	E6, E7, E13, E20, E29, E30, E31, E36, E37, E42, E53, E54, E55, E79, E80, E86, E87, E93, E104, E105, E106, E111, E112, E118
Right temporal (RT)	E92, E96, E97, E98, E101, E102, E103, E108, E109, E110, E115, E116, E117, E122
Posterior (P)	E59, E60, E61, E62, E65, E66, E67, E70, E71, E72, E75 E76, E77, E78, E83, E84, E85, E90, E91,

The channels in Table 3 are channels of interest, while the other channels are marginal and do not belong to the brain regions classified above.

**FIGURE 5:**
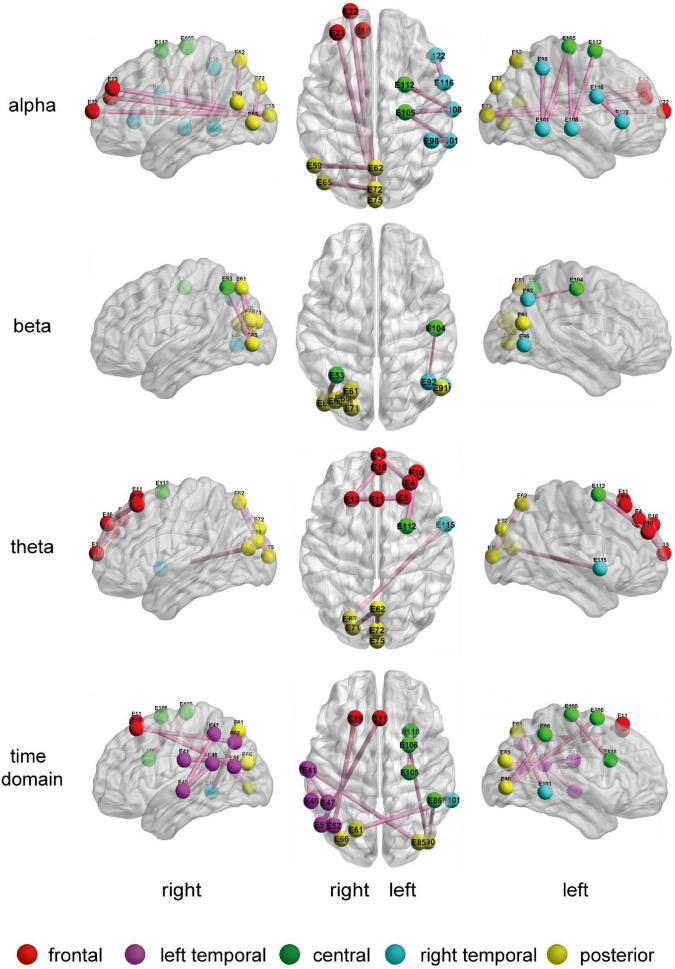
The 10 most frequently selected connection diagrams for different LO-BFNs.

Figure 5 shows the 10 most discriminative FC feature maps for the different LO-BFNs. The node color indicates the brain region the channel belongs to, the connection line represents the correlation between two channels, and the line width indicates the frequency. The thicker line represents the higher frequency. It can be seen that the frequently selected FC features in LO-BFN often appear in the LF, RC, RT, and LP regions in the alpha band; left central-right central (LC-RC), RT and left posterior-right posterior (LP-RP) regions in the beta band; the left frontal-right frontal (LF-RF), RC, RT, and LP regions in the theta band, and the LF, RC, left temporal-right temporal (LT-RT) and left posterior-right posterior (LP-RP) regions in the time domain.

**FIGURE 6:**
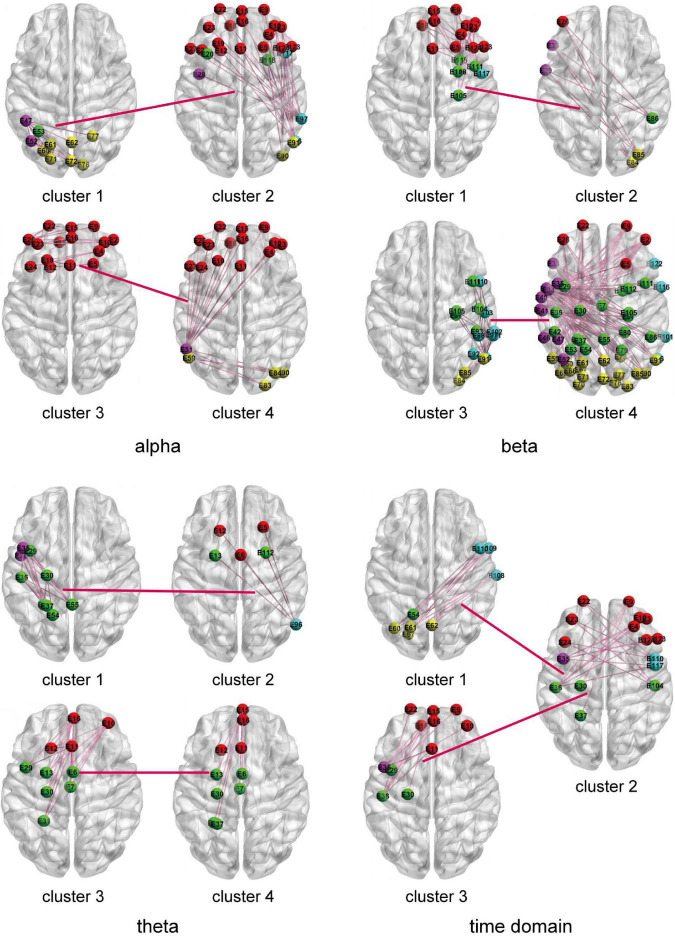
The two most frequently selected connection diagrams for different HO-BFNs. Each brain diagram represents the FC of interest channels across the brain region in a cluster.

The HO-BFN is constructed based on clustering. Each cluster is used as the vertex of the network, and the HO-FC is used as the edge of the network. For HO-BFN, we select the two most discriminative cluster pairs and describe the FC feature diagram between these two cluster pairs in Figure 6, where each brain map represents a cluster, the connection between brain maps represents the connection between clusters, that is, HO-FC, and the connections in the brain map represent the FC between channels belonging to this cluster, that is, LO-FC. Table 4 shows the brain regions involved in the most discriminative clusters that are selected from different HO-BFNs. From Figure 6 and Table 4, we can observe that the most frequently selected features in HO-BFN are mainly distributed in LF-RF, LT-RT, and LC-RC regions of the theta band, other brain regions except RT in the time domain, as well as the whole brain region of the alpha and beta band.

**TABLE 4:** The brain regions selected from different HO-BFNs.

Cluster	Alpha	Beta	Theta	Time domain
Cluster 1	LT, LC, LP-RP	LF-RF, RC, RT	LT, LC	LC, LP, RT
Cluster 2	LF-RF, LT-RT, LC-RC, RP	LF, LT, RC, RP	LF-RF, LC-RC, RT	LF-RF, LT-RT, LC-RC
Cluster 3	LF-RF	RC, RT, RP	LF-RF, LC	LF-RF, LT, LC
Cluster 4	LF-RF, LT, LP-RP	LF-RF, LT-RT, LC-RC, LP-RP	LF, LC	LF-RF, LT-RT, LC-RC

## Discussion

In this paper, we propose a novel method to construct HO-BFN based on the “correlation’s correlation” strategy, which can simultaneously capture low-order features reflecting FC information between any two channels and high-order features reflecting FC information among multiple channels, so as to better simulate the mechanisms of the deep brain and provide more discriminative information for the diagnosis of mental disorders. We believe that different BFNs can mine disease-disturbed BFN variation information from different aspects, which has better performance in MDD classification experiments. According to the experimental results, we will discuss HO-BFNs in more detail.

In the experiment to explore the influence of the clustering parameter on the classification accuracy of HO-BFN, we find that the classification result changes with the change of the clustering parameter. When the clustering parameter is too small or too large, the accuracy of diagnosis will gradually decrease. This can be understood from two aspects: (1) when the clustering parameter is too small, the LO-FC time series with different dynamic changes may be divided into the same cluster, which will reduce the similarity of each cluster, so that the HO-BFN constructed by the mean sequence of each class as the vertex is unreliable; and (2) when the clustering parameter is too large, the LO-FC time series with similar dynamic changes may be divided into different clusters, which will increase the number of features extracted from HO-BFN, thus resulting in more redundant features and causing the decrease in accuracy and generalization ability. Thus, choosing the suitable number of clusters for HO-BFN is the key to achieving MDD classification and improving the classification accuracy.

According to the experimental results in Table 2, we find that the proposed BFNs in the frequency domain are usually more discriminative than in the time domain, and the alpha and theta bands in the frequency domain are more discriminative than the beta band, indicating that the brain functional structure of MDD patients has undergone significant changes in these two bands. Therefore, we believe that the BFNs of alpha and theta play an important role in the pathogenesis of MDD. Several previous studies have also reported the same or similar conclusion: abnormal brain function in MDD patients occurs at certain frequency bands. For example, Fingelkurts et al. (2010) showed that the FC in the alpha and theta bands of EEG in MDD was impaired. Hosseinifard et al. (2013) found that there were differences in alpha bands between the MDD group and the NC group.

To further prove the effectiveness of BFNs in MDD disease diagnosis, we trace the BFNs to which the features of the classifier used for training belong. The experimental results in Figures 5, 6 show that: (1) the channel pairs selected by LO-BFN hardly overlap with those of HO-BFN, indicating that the FC features extracted from LO-BFN and HO-BFN are complementary to each other. (2) RC and RT are significantly different in all LO-BFNs, and they are related to the regulation of attention, long-term memory, and emotion. In previous studies, Fan et al. (2013) found that abnormal RT superior gyrus activity could be a potential marker of suicidal tendencies in MDD patients. Sun et al. (2019) observed differences in MDD patients in the F, T, and C of the theta band and in the T and C of the alpha band. Zhang et al. (2021) found significant modifications in brain synchrony of LF, T, and RT in MDD patients. Our observations are generally consistent with these studies (Fan et al., 2013; Sun et al., 2019; Zhang et al., 2021).

However, this study has some limitations. Firstly, either the low-order or the high-order FC is based on the correlation instead of the inherent causality. The relatively small sample size and unbalanced data may also affect the result of the analysis. Although most conclusions obtained by our method are generally consistent with the previous relevant studies, the experimental results may have a potential bias due to the heterogeneity of the experiment (Hassler and Thadewald, 2010). In the future work, we will focus on the influence of nonsensical and biased correlation and investigate more advanced models to calculate correlation, such as causality inference, based on larger datasets to obtain more accurate and sufficient information about the brain changes of MDD patients. Secondly, the sliding window algorithm should set the step size and window width, but we fixed the window width and step length in this study. Further studies will investigate the influence of different parameter settings. Finally, we linearly combine the decision scores of the LO-BFN and HO-BFN at the decision-making level, and this linear combination may not fully mine the complementary information, thereby affecting the classification accuracy. Therefore, in our future work, we will further increase the accuracy of MDD diagnosis by using more advanced information fusion strategies.

## Conclusion

In this paper, we propose a framework for constructing HO-BFN based on the “correlation’s correlation” strategy and capture the high-order correlations across different channels for MDD diagnosis. We use hierarchical clustering to reduce the computational complexity of the HO-BFN. Experimental results demonstrate that: (1) the proposed HO-BFN can provide discriminative information for the MDD identification. (2) Fusing high-order and low-order BFNs can significantly improve the recognition rate of MDD patients. (3) The most discriminative brain regions are associated with the regulation of attention, and these findings are consistent with the daily behavior of MDD patients.

## Data availability statement

Publicly available datasets were analyzed in this study. This data can be found here: http://modma.lzu.edu.cn/data/index/.

## Author contributions

FZ: conceptualization, methodology, writing – review, and editing. HP: conceptualization, software, writing – original draft, methodology, formal analysis, investigation, and validation. NL: validation. XC, HZ, NM, and YR: writing – review, and editing. All authors contributed to the article and approved the submitted version.
